# Supplementary material for: Genome-wide discovery and integrative genomic characterization of insulin resistance loci using serum triglycerides to HDL-cholesterol ratio as a proxy
Source: Nat Commun. 2024 Sep 14;15:8068. doi: 10.1038/s41467-024-52105-y (PMC11401929; doi:10.1038/s41467-024-52105-y)
Supplement: Supplementary file 1 — Supplementary Information [file 41467_2024_52105_MOESM1_ESM.pdf]

A

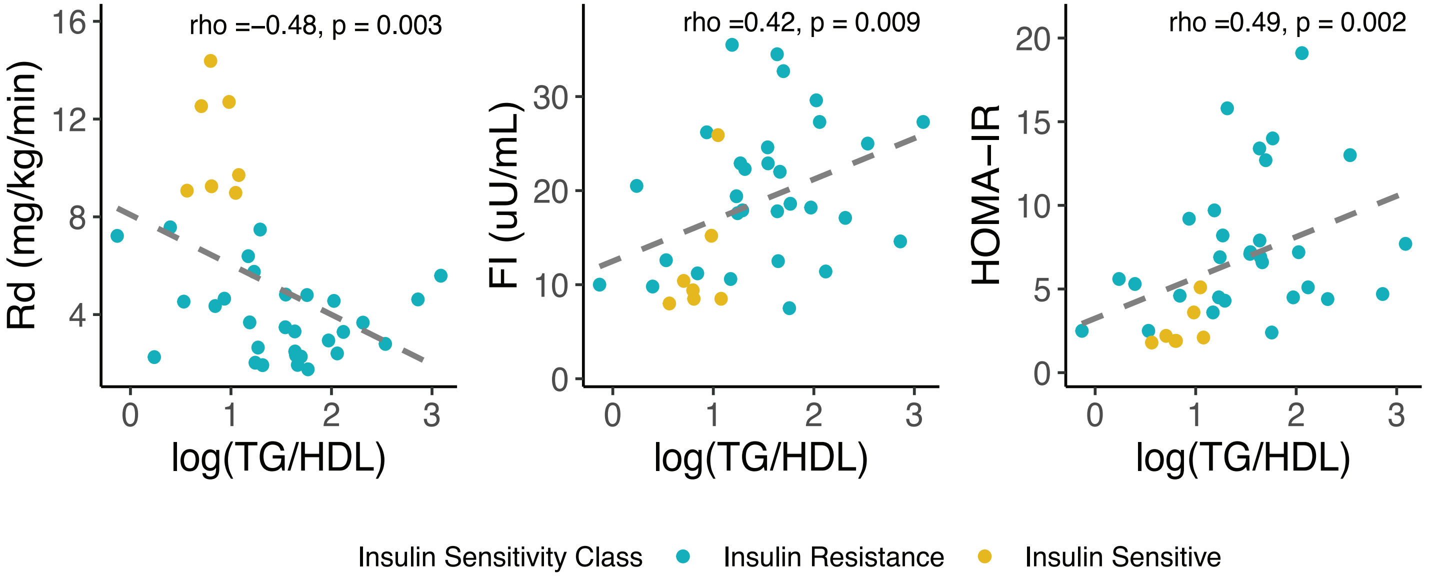

B

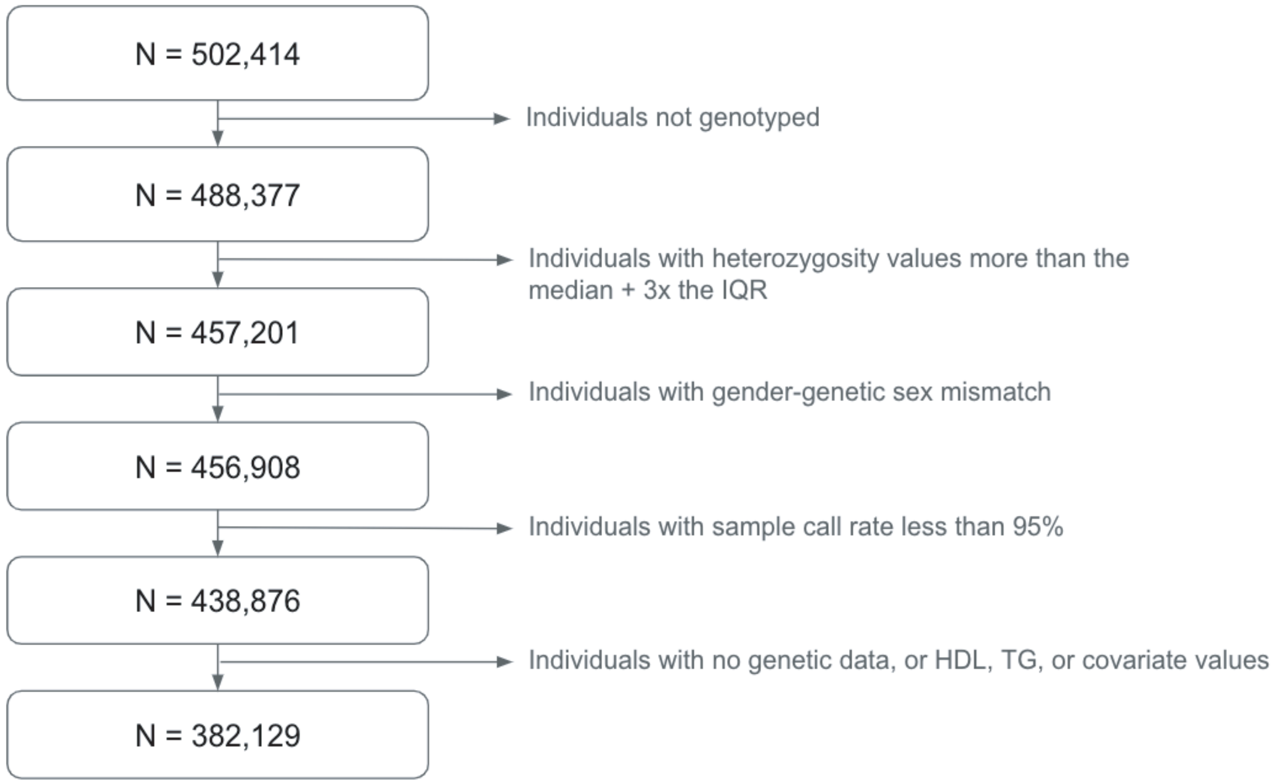

Supplementary Figure 1. Correlation of TG/HDL with other measures of insulin resistance and participant-level filtering in the UK Biobank.

A) Spearman correlations among 45 individuals between TG/HDL and measures of insulin resistance including glucose disposal rate (Rd) measured by undergoing hyperinsulinemic-euglycemic clamp (left), fasting insulin (FI, middle), and Homeostatic Model Assessment for Insulin Resistance (HOMA-IR, right). Spearman rank correlation coefficients (rho) and p-values are given. Insulin resistance refers to Rd > 8 mg/kg/min. B) The number of participants in the UK Biobank are listed after each level of quality control. N = sample size; “covariate” includes age, sex, and 1-20 PCs.

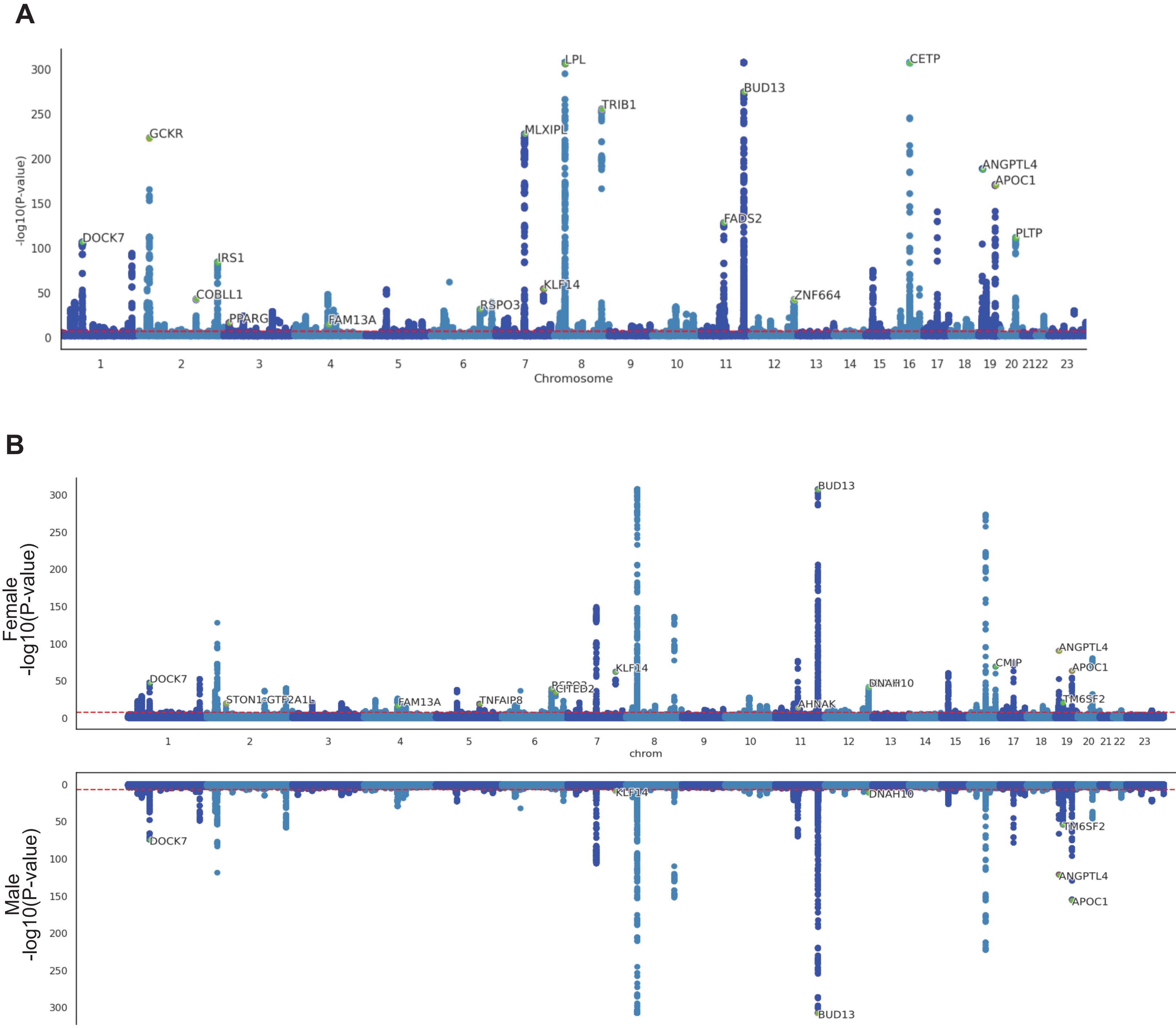

Supplementary Figure 2. GWAS for TG/HDL identifies 251 TG/HDL genomic risk loci and 17 sex-dimorphic loci.

A) Manhattan plot of GWAS for TG/HDL. Nearest gene to the lead SNP is labeled for known insulin resistance loci.

B) Sex-stratified GWAS for TG/HDL. Nearest gene to the lead SNP is labeled for the 17 sex-dimorphic TG/HDL risk loci identified (sex-dimorphic p-value < 0.05 / 251).

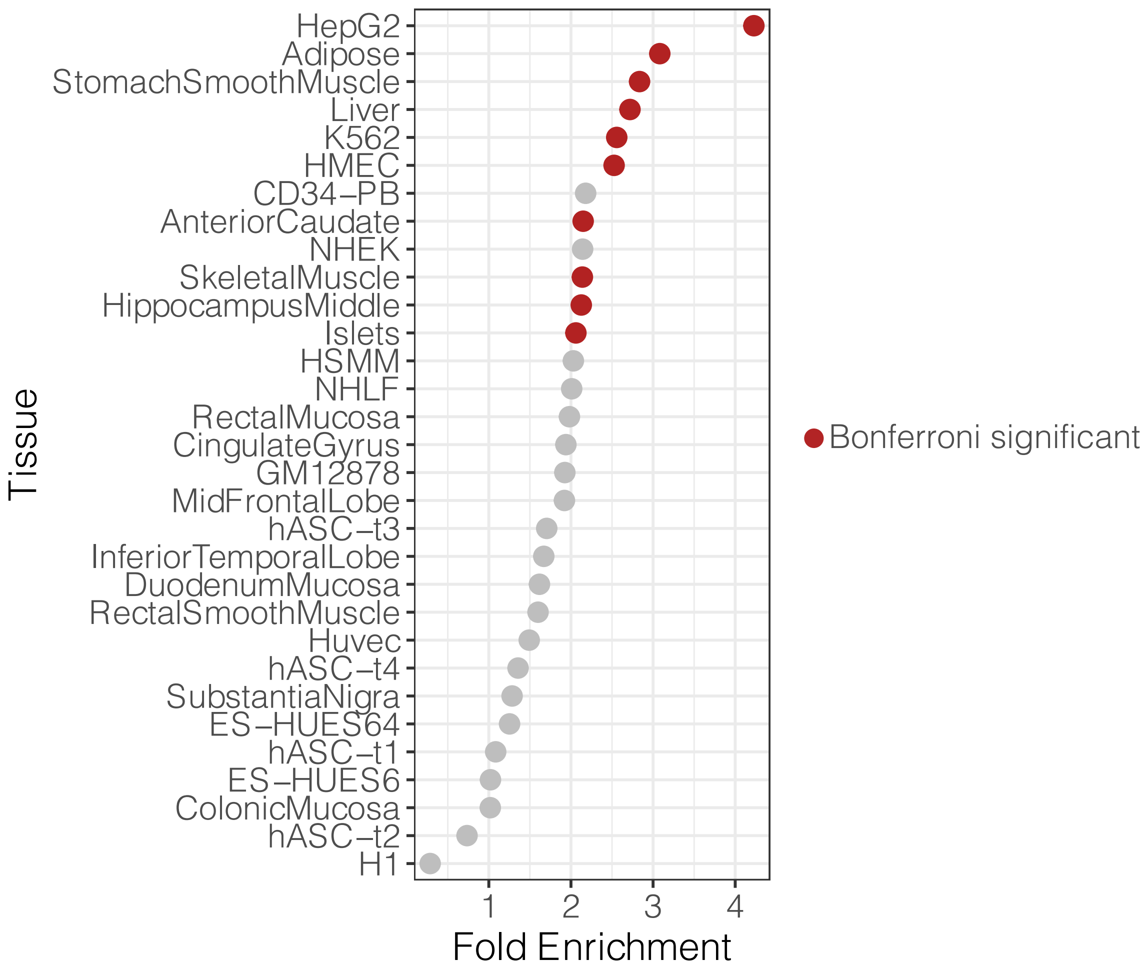

Supplementary Figure 3. TG/HDL genomic risk loci are highly enriched in metabolically implicated tissues.

Tissue enrichment for the TG/HDL genomic risk loci as determined by GREGOR. Tissues with significant enrichment signals in tissue-specific regulatory elements upon Bonferroni correction ($p < 0.05 / 31$ tissues) are highlighted in red.

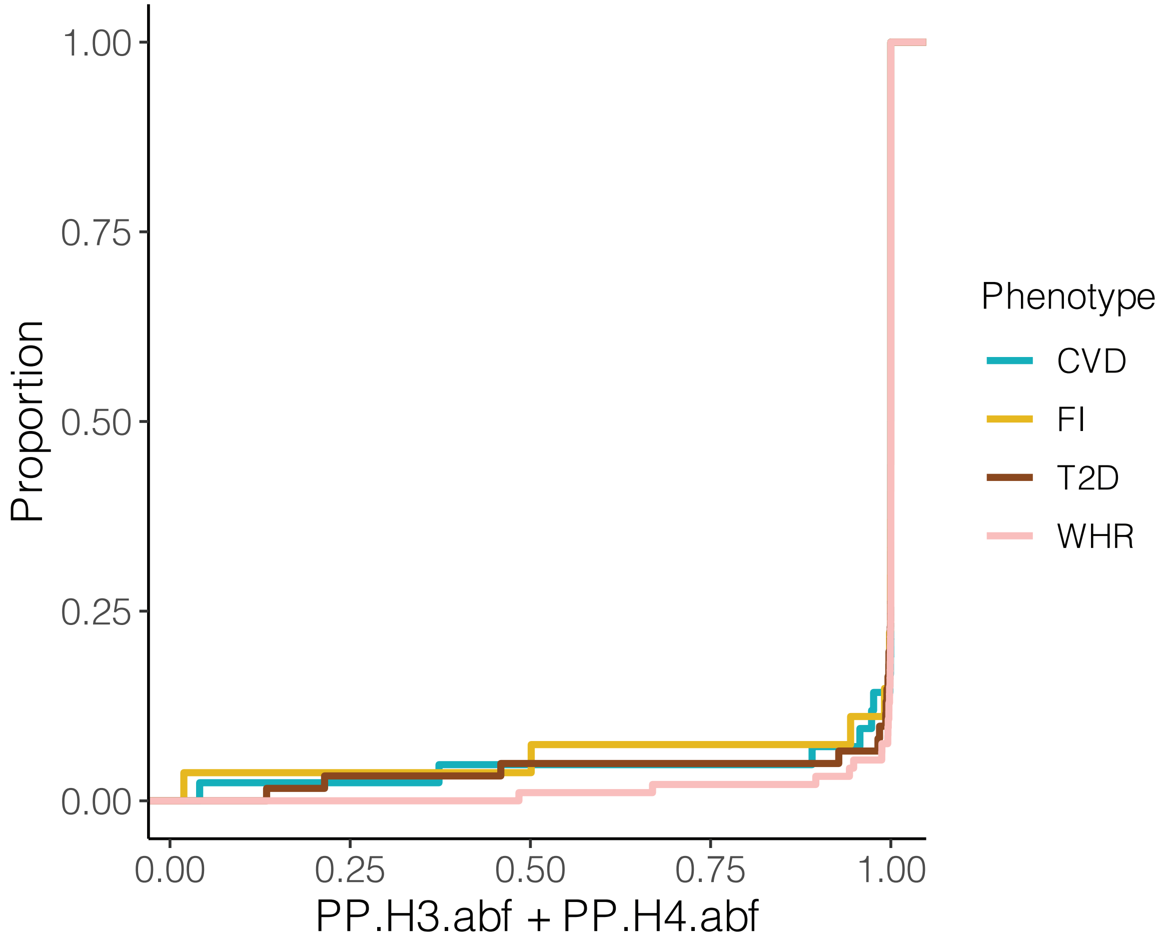

Supplementary Figure 4. Genetic colocalization between TG/HDL association signals and insulin resistance related phenotypes.

Cumulative distribution plot of sum of Posterior Probability Bayesian factor H3 and H4 (PP.H3.abf+PP.H4.abf) for genetic colocalization between 251 TG/HDL associated loci with association signals from waist-hip ratio (WHR), type 2 diabetes (T2D), cardiovascular disease (CVD), and fasting insulin (FI). Over 90% of signals have PP.H3.abf+PP.H4.abf ≥ 0.99 indicating a high degree of genetic overlap with TG/HDL.
