# Supplementary material for: Genome-wide discovery and integrative genomic characterization of insulin resistance loci using serum triglycerides to HDL-cholesterol ratio as a proxy
Source: Nat Commun. 2024 Sep 14;15:8068. doi: 10.1038/s41467-024-52105-y (PMC11401929; doi:10.1038/s41467-024-52105-y)
Supplement: Supplementary file 3 — Description of Additional Supplementary Files [file 41467_2024_52105_MOESM3_ESM.pdf]

Description of Additional Supplementary Files

File name: Supplementary Data 1
Description: 251 Genomic risk loci determined from GWAS for TG/HDL in the UK Biobank. Each locus is defined by a "Lead SNP", and "Nearest Gene" indicates the closest gene to the lead SNP. Chromosome and genomic positions (Chr:Pos) of the lead SNP are according to GRCh37. Lead SNP allele frequency (EAF), betas, and standard errors (SE) are in reference to the Effect Allele. Indicated locus P-value ("P-value (corrected)") determined by the lead SNP is corrected for genomic inflation. Listed are the -log10(P-values) of the lead SNP from GWAS for TG and HDL individually which were used to compute the locus boost score (TG/HDL Boost Statistic). Per locus sex-dimorphic t-statistics and p-values computed from sex-stratified GWAS for TG/HDL are given, and "Sex-dimorphic locus" = Y indicates if the locus is significantly sex-dimorphic (Sex-dimorphic p-value < 0.05/251). "MGBB Replication SNP" represents the proxy SNP used for replication in the MGBB cohort if the lead SNP was not available, and NA indicates no proxies for that locus were able to be assessed in the MGBB. The TG/HDL association beta and p-value of the lead SNP / replication SNP in the MGBB are given, along with the calculated power for replication for each lead SNP / replication SNP.

File name: Supplementary Data 2
Description: TG/HDL associated loci identified from sex-stratified GWAS in the UK Biobank not present in sex-combined GWAS. Each locus is defined by a "Lead SNP", and "Nearest Gene" indicates the closest gene to the lead SNP. Chromosome and genomic positions (Chr:Pos) of the lead SNP are according to GRCh37. Lead SNP allele frequency (EAF), betas, and standard errors (SE) are in reference to the Effect Allele. Indicated locus P-value ("P-value (corrected)") determined by the lead SNP is corrected for genomic inflation. Listed are the -log10(P-values) of the lead SNP from the sex-stratified GWAS for TG and HDL individually which were used to compute the locus boost score (TG/HDL Boost Statistic) for the listed sex.

File name: Supplementary Data 3
Description: Genetic correlations of TG/HDL with insulin resistance biomarkers and outcomes computed using cross-trait LD Score (LDSC) regression. Pairs of phenotypes correlated are indicated as "Pheno" 1 and 2. The coefficient of genetic correlation (rg) and standard error (se) are listed, along with the z-score (z) and p-value (p). Right table details the GWAS summary statistics used in correlations.

File name: Supplementary Data 4
Description: Overlap of 251 TG/HDL loci with previously identified insulin resistance genomic signals. TG/HDL locus lead SNP, chromosome, and locus genomic regions (TG/HDL locus start:end) are indicated. Significant SNPs from previous studies which overlap with a TG/HDL risk locus are listed along with the corresponding phenotype (WHR, waist-hip ratio; T2D, type 2 diabetes; FI/IR, fasting insulin/HOMA-IR; CVD, coronary artery disease). The corresponding beta, standard error (SE), and p-value (P) for each SNP in the indicated previous study is given. “No_overlap” is listed if no significant SNPs from the previous insulin resistance related GWAS

fell within the locus boundaries of a TG/HDL genomic risk locus identified in our GWAS. Loci not previously identified in other studies are marked "novel".

File name: Supplementary Data 5
Description: Genetic colocalization of the 251 TG/HDL risk loci with association signals from insulin resistance related phenotypes: waist-hip ratio (WHR), type 2 diabetes (T2D), coronary artery disease (CVD), and fasting insulin (FI). TG/HDL locus lead SNP, chromosome, and locus genomic regions (TG/HDL locus start:end) are indicated. For each significant SNP that positionally overlapped with the corresponding phenotype, the R^2 value, the number of SNPs, and the posterior probabilities are listed. Colocalization posterior probabilities for hypotheses H0–H4 (abbreviated as "PP.H0.abf", "PP.H1.abf", "PP.H2.abf", "PP.H3.abf", and "PP.H4.abf"): H0 = no association with either TG/HDL or the listed phenotype, H1 = association with TG/HDL only, H2 = association with the listed phenotype only, H3 = association with both TG/HDL and the list phenotype with 2 independent SNPs at locus, H4 = association with both TG/HDL and the list phenotype with 1 shared SNP.

File name: Supplementary Data 6
Description: Fine-mapping results for the 50 top quartile boosted TG/HDL risk loci which genes were nominated for. The lead SNP defining the TG/HDL risk locus is shown. The 95% credible sets are indicated, and each credible set per locus is assigned a unique numerical label (ie. credible set #1, #2,... #n). The SNPs within each credible set are listed along with the GWAS betas, standard errors (SE), and p-values. The PIP for each SNP in the credible set determined by fine-mapping is given. If a SNP has a PIP > 0.1 and has a genomic annotation (i.e. exonic variant, eQTL) used to nominate the causal gene, the annotation is noted in "Annotation description". If no annotations were available for any causal SNPs in the credible sets, the SNP with the highest PIP used to nominate the nearest gene is indicated in "Annotation description" along with the distance of the SNP to the nominated gene.

File name: Supplementary Data 7
Description: Genomic evidence for nominated TG/HDL associated genes to determine directionality and tissue-specificity. Locus lead SNPs and nominated genes are given. SNPs within a 95% credible set which are either exonic variants or eQTLs are listed along with the corresponding TG/HDL GWAS beta and fine-mapping PIP. If the fine-mapped exonic SNP is a missense variant, the protein change is listed ("Exonic variant") and the predicted function is indicated for "PolyPhen-2" and "SIFT". If the fine-mapped SNP is an eQTL, the study from which the eQTL was identified is listed ("eQTL"). Beta and p-values from loss-of-function burden tests ("pLOF Burden") for each gene in the UK Biobank extracted from Genebass are noted. Association statistics (t-statistics (T), p-values (P)) of heritable gene expression with TG/HDL in the UK Biobank for metabolic tissues (subcutaneous (subc) adipose, visceral adipose (visc), liver, and muscle)) are noted.

File name: Supplementary Data 8
Description: Gene expression (transcripts per million, TPM) per tissue for genes of interest in the GTEx study.

File name: Supplementary Data 9
Description: PLA2G12A loss-of-function (LOF) burden tests in the UK Biobank exome sequenced samples with different definitions for LOF variants (masks). For each level of mask and method (standard burden test, excluding the top 2 causal variants, or conditioning on the top 2 causal variants), the betas, standard errors (SE), pvalues, the number of variants in mask, and the number of variant carriers are provided. The LOF variants (masks) are defined as: 5of5 = variants predicted deleterious by all five prediction tools, 1of5_1pct = rare (MAF < 1%) variants predicted deleterious by at least 1 tool, 0of5_1pct = all rare variants (MAF < 1%).

File name: Supplementary Data 10
Description: Differential expression analysis in biopsied skeletal muscle of 35 individuals upon TZD treatment (post-TZD vs pre-TZD; PMID: 19841271). For each gene and Ensembl gene ID (ENSG), the log2 fold changes (log2FC) , t-statistics (t) , P-values, and adjusted P-values (adj.P.Val) are listed.

File name: Supplementary Data 11
Description: Correlation between glucose disposal rate (Rd) and adipose tissue gene expression. Adipose gene expression levels were regressed on glucose disposal rate measured by hyperinsulinemic-euglycemic clamp in the 35 individuals using linear regression. For each gene and Ensembl gene ID (ENSG), the estimates, standard errors (SE), and P-values are listed.

File name: Supplementary Data 12
Description: Results of regression for metabolic traits including TG/HDL against serum TNFAIP8 levels extracted from Olink explore proteomic data obtained on UK Biobank participants. the combined models are corrected for age and sex and the sex-specific models are corrected for age. For each trait and sex stratification, the betas, standard errors (SE), P-values, and the number of individuals analyzed (n) are listed.

File name: Supplementary Data 13
Description: GWAS summary statistics for TNFAIP8 locus lead SNP rs1045241 across metabolic disease phenotypes for which sex-stratified association studies have been conducted. For each phenotype and sex, the GWAS betas, standard errors (SE), and pvalues are given, and the number of individuals in each analysis is listed.
