# Supplementary material for: Genome-wide discovery and integrative genomic characterization of insulin resistance loci using serum triglycerides to HDL-cholesterol ratio as a proxy
Source: Nat Commun. 2024 Sep 14;15:8068. doi: 10.1038/s41467-024-52105-y (PMC11401929; doi:10.1038/s41467-024-52105-y)
Supplement: Supplementary file 5 — Reporting Summary [file 41467_2024_52105_MOESM5_ESM.pdf]

nature portfolio

Corresponding author(s): Amit Majithia

Last updated by author(s): Jul 18, 2024

Reporting Summary

Nature Portfolio wishes to improve the reproducibility of the work that we publish. This form provides structure for consistency and transparency in reporting. For further information on Nature Portfolio policies, see our Editorial Policies and the Editorial Policy Checklist.

Statistics

For all statistical analyses, confirm that the following items are present in the figure legend, table legend, main text, or Methods section.

n/a	Confirmed	
☐	☒	The exact sample size (*n*) for each experimental group/condition, given as a discrete number and unit of measurement
☐	☒	A statement on whether measurements were taken from distinct samples or whether the same sample was measured repeatedly
☐	☒	The statistical test(s) used AND whether they are one- or two-sided<br>*Only common tests should be described solely by name; describe more complex techniques in the Methods section.*
☐	☒	A description of all covariates tested
☐	☒	A description of any assumptions or corrections, such as tests of normality and adjustment for multiple comparisons
☐	☒	A full description of the statistical parameters including central tendency (e.g. means) or other basic estimates (e.g. regression coefficient) AND variation (e.g. standard deviation) or associated estimates of uncertainty (e.g. confidence intervals)
☐	☒	For null hypothesis testing, the test statistic (e.g. *F*, *t*, *r*) with confidence intervals, effect sizes, degrees of freedom and *P* value noted<br>*Give P values as exact values whenever suitable.*
☒	☐	For Bayesian analysis, information on the choice of priors and Markov chain Monte Carlo settings
☒	☐	For hierarchical and complex designs, identification of the appropriate level for tests and full reporting of outcomes
☐	☒	Estimates of effect sizes (e.g. Cohen's *d*, Pearson's *r*), indicating how they were calculated

Our web collection on statistics for biologists contains articles on many of the points above.

Software and code

Policy information about availability of computer code

Data collection	No software was used for data collection
Data analysis	Public software used for data analysis include REGENIE, Ensembl Variant Effect Predictor (VEP), PLINK2, GREGOR, LDSC, FUMA, SuSie, COLOC, Kallisto, Dream, and R.

For manuscripts utilizing custom algorithms or software that are central to the research but not yet described in published literature, software must be made available to editors and reviewers. We strongly encourage code deposition in a community repository (e.g. GitHub). See the Nature Portfolio guidelines for submitting code & software for further information.

Data

Policy information about availability of data

All manuscripts must include a data availability statement. This statement should provide the following information, where applicable:

- Accession codes, unique identifiers, or web links for publicly available datasets
- A description of any restrictions on data availability
- For clinical datasets or third party data, please ensure that the statement adheres to our policy

Full GWAS summary statistics from this study are available at the GWAS Catalog (https://www.ebi.ac.uk/gwas/) under study accession codes GCST90435481, GCST90435482, GCST90435483. Individual level genomic and phenotypic data from the UK Biobank are available to researchers upon application (https://ukbiobank.ac.uk). The summary statistics of previous insulin resistance related GWAS used in this study are available as described in Supplementary Data 3. The

functional genomic annotations for SNP to gene linking used in this study were downloaded (November 2023) from https://alkesgroup.broadinstitute.org/cS2G. The Genebass exome-based association statistics in the UK Biobank (accessed November 2023) are available here: https://app.genebass.org/. The Functional Summary-based Imputation (FUSION) GTEx v8 multi-tissue expression statistics used in this study were downloaded (November 2023) from http://gusevlab.org/projects/fusion/#gtex-v8-multi-tissue-expression. The metabolic disease/trait associations meta-analyzed in this study (accessed November 2023) were obtained from the Common Metabolic Diseases Knowledge Portal (cmdkp.org) https://hugeamp.org/variant.html?variant=rs1045241. The ChIP-seq of PPARG in human adipose stem cells67 and estrogen receptor (ESR1) in human breast cell line ZR-75-169 used in this study are available in the GEO under accession codes GSM534493 and GSM798427 respectively. All other data are available within the article or from the corresponding author upon request.

Research involving human participants, their data, or biological material

Policy information about studies with human participants or human data. See also policy information about sex, gender (identity/presentation), and sexual orientation and race, ethnicity and racism.

Reporting on sex and gender	Genome-wide association analyses were performed including all individuals as well as in a sex-stratified manner. Sex was defined using UK Biobank data-field 31.
Reporting on race, ethnicity, or other socially relevant groupings	To correct for population stratification, all analyses were adjusted for the first 20 principal components of ancestry.
Population characteristics	~500,000 individuals aged 40-69 as detailed in Bycroft et al. Nature 2018
Recruitment	Volunteers across the UK
Ethics oversight	UK Biobank

Note that full information on the approval of the study protocol must also be provided in the manuscript.

Field-specific reporting

Please select the one below that is the best fit for your research. If you are not sure, read the appropriate sections before making your selection.

☒ Life sciences ☐ Behavioural & social sciences ☐ Ecological, evolutionary & environmental sciences

For a reference copy of the document with all sections, see nature.com/documents/nr-reporting-summary-flat.pdf

Life sciences study design

All studies must disclose on these points even when the disclosure is negative.

Sample size	Sample size chosen by number of participants available in the UK Biobank.
Data exclusions	Sample level filters to the UK Biobank participants were applied following the GLGC standards, detailed in the manuscript methods.
Replication	Findings replicated in the independent cohort, Mass General Biobank
Randomization	Genetic association: Participants randomized by nature with covariate corrections performed
Blinding	Blinding was not relevant to this study

Reporting for specific materials, systems and methods

We require information from authors about some types of materials, experimental systems and methods used in many studies. Here, indicate whether each material, system or method listed is relevant to your study. If you are not sure if a list item applies to your research, read the appropriate section before selecting a response.

Materials & experimental systems

n/a	Involved in the study	
☒	☐	Antibodies
☒	☐	Eukaryotic cell lines
☒	☐	Palaeontology and archaeology
☒	☐	Animals and other organisms
☐	☒	Clinical data
☒	☐	Dual use research of concern
☒	☐	Plants

Methods

n/a	Involved in the study	
☐	☒	ChIP-seq
☒	☐	Flow cytometry
☒	☐	MRI-based neuroimaging

Clinical data

Policy information about clinical studies

All manuscripts should comply with the ICMJE guidelines for publication of clinical research and a completed CONSORT checklist must be included with all submissions.

Clinical trial registration	Clinical study: https://doi.org/10.1073/pnas.0903032106
Study protocol	Described in previous study: https://doi.org/10.1073/pnas.0903032106
Data collection	Described in previous study: https://doi.org/10.1073/pnas.0903032106
Outcomes	Described in previous study: https://doi.org/10.1073/pnas.0903032106

Plants

Seed stocks	N/A
Novel plant genotypes	N/A
Authentication	N/A

ChIP-seq

Data deposition

☒ Confirm that both raw and final processed data have been deposited in a public database such as GEO.

☒ Confirm that you have deposited or provided access to graph files (e.g. BED files) for the called peaks.

Data access links<br>*May remain private before publication.*	Data are deposited in the Gene Expression Ominibus under accession number GSE32222. GSM798427 results were used in this study and can be found at https://chip-atlas.org/view?id=SRX371471
Files in database submission	62 samples: https://www.ncbi.nlm.nih.gov/geo/query/acc.cgi?acc=GSE32222
Genome browser session<br>(e.g. UCSC)	*Provide a link to an anonymized genome browser session for "Initial submission" and "Revised version" documents only, to enable peer review. Write "no longer applicable" for "Final submission" documents.*

Methodology

Replicates	Described in previous study: https://www.nature.com/articles/nature10730
Sequencing depth	Described in previous study: https://www.nature.com/articles/nature10730
Antibodies	Described in previous study: https://www.nature.com/articles/nature10730
Peak calling parameters	Determined by ChIP Atlas central data processing pipeline.
Data quality	Determined by ChIP Atlas central data processing pipeline.
Software	IGV v2.8.2 for visualization
